# Energy efficiency: The evolution of a motherhood concept

**DOI:** 10.1177/03063127221096171

**Published:** 2022-06-09

**Authors:** Tessa Dunlop

**Affiliations:** DG Joint Research Centre, European Commission, Ispra, Italy

**Keywords:** energy efficiency, climate policy, energy policy

## Abstract

Energy efficiency is a popular policy strategy to reduce energy consumption and greenhouse gas emissions. The concept of energy efficiency is relatively simple – to use less energy for the same level of output. However, complexities emerge when applying efficiency concepts to real world processes and practices of energy consumption: subjective judgments when defining, measuring and applying energy efficiency principles, how efficiency is conceptualized and applied in policy, and how policy is designed and implemented, given social and environmental tradeoffs. This article traces the evolution of EU energy efficiency policy over seven decades to better understand underlying values and tradeoffs from a sociological perspective. Using insights from critical policy studies, the article reveals how certain values are reflected in how energy efficiency is defined and measured over time. It highlights how the conceptualization of energy efficiency has been used as an effective rhetorical device – and how some potentially relevant concepts and issues get sidelined in favor of others. The analysis illustrates how narrow conceptualizations of energy efficiency has put blinders on wider environmental and social issues. This points to the need for a more nuanced policy approach that takes into account the complexities and uncertainties of societal and policy challenges. The findings point to the need for energy efficiency policy that pays closer attention to citizens’ views and collective solutions in order to formulate more effective policy to reduce energy consumption.

## The problem

In broad terms, energy efficiency is a straightforward concept – to use less energy for the same level of output. Dig a little deeper, however, and it proves to be a complex idea, imbued with multiple meanings (Dunlop, 2019). The complexity in the definition of energy efficiency stems from its ambiguity. Energy efficiency indicators are generally materialized as a ratio with a numerator and denominator that represent an input and output of energy – that is, measuring how much energy input it takes to produce an output. The input is generally a source of energy, such as electricity or gas. The output, however, is whatever is deemed ‘useful energy output’, such as lighting, heat or transport. However, it is a major problem to determine what a ‘useful’ output is (Patterson, 1996), because ‘useful’ is subjective and thus contains built-in value judgments. As Boulding (1981: 153) writes, ‘The significance of the efficiency concept … depends on the significance of the outputs and inputs in terms of human valuations’.

This problem affects evaluations of efficiency more broadly. Wildavsky (2018) observes that efficiency ‘does not tell you where to go, but only that you should arrive there (or go part of the way) with the least effort’. Stone (2012) defines efficiency as ‘getting the most for the least, or achieving an objective for the lowest cost’ (Stone, 2012). Due to its lack of concrete values, she does not see efficiency as a goal in and of itself, but as a means to achieve different goals. The analysis here applies Stone’s (2012) approach to efficiency, using in particular the concept of ‘motherhood issues’ to understand the underlying values inherent in the conceptualization of energy efficiency in EU policy.

### Energy efficiency as a motherhood issue

‘Post-positivist policy analysis’ treats policymaking as a dynamic space where ideas and values are constantly contested. Under this umbrella, the ‘argumentative turn’ (Fischer and Forester, 1993; Majone, 1992) puts language and argumentation at the center of the policy process. As Hajer and Versteeg (2005) state: ‘Language profoundly shapes one’s view of the world and reality, instead of being only a neutral medium mirroring it’ (p. 176). Drawing on Foucault, Dryzek (2013: 9) sees discourse as ‘a shared way of apprehending the world’. Here, policy language is analyzed to understand the critical issues of truth and power in the construction of causality, legitimacy and responsibility, interests, needs, values, preferences and obligations.

Stone (2012) contends that policymaking is a constant discursive struggle over problem definitions, societal classification, category boundaries and the definition of ideas, which guide how people behave. The conceptual framings of problems and goals determine ideas about how these problems should be solved and whose responsibility it is to solve them. Policymaking involves disputes over ‘motherhood issues’ – enduring values of community life that are the standards of analysis most commonly invoked in policy debates, such as equity, efficiency, welfare, liberty, and security. While generally supported by everyone in the abstract, these motherhood issues often lead to contradictory interpretations when concretized. ‘Behind every policy issue lurks a contest over conflicting, though equally plausible, conceptions of the same abstract goal or value’ (Stone, 2012: 14). Therefore, ideals often conflict in policy implementation, necessitating compromise. One task of the political analyst is then to clarify the underlying value disputes to see where they differ, so that differing parties are able to move toward some reconciliation.

To avoid the paradoxical trap of the ‘rationality project’, rendering policy apolitical through the application of rational, managed and simplified analytical methods, Stone proposes a mode of policy analysis that recognizes the complex, messy, and ambiguous nature of policy due to the multiple values and perspectives involved. This involves taking stock of analytical concepts, problem definitions and policy instruments as political claims in themselves, instead of granting them privileged status as universal truths. In particular, she highlights the importance of analyzing the complexity of problem definition to understand the multiple conceptions of the given motherhood issue. Therefore, politics should be viewed as a way for people to help each other see from different perspectives – a desirable process that can allow society to avoid myopia and help solve common problems more effectively. In this process, values matter – and policy analysts and decision makers must bring their own values into the picture. To understand the underlying values in efficiency policy, Stone identifies three main areas of contention that should be highlighted: Who gets the benefits and bears the burdens of a policy? How should we measure the benefits and costs of a policy? And what mode of organizing human activity is likely to yield the most effective results?

This article approaches energy efficiency as co-constructed in that the conceptualization of energy efficiency depends on various actors, institutional logics and discourses about its use and implementation. It represents a solution that depends on the specific creation of multiple problem definitions. Meanings are created, argued and won through language and discourse in politics, and some potentially relevant concepts get sidelined in favor of others. I apply this frame to understand how energy efficiency is used as a rhetorical device in EU policy and to see the sociological and environmental problems embedded within seemingly neutral technical definitions and measures. I apply Stone’s discursive toolbox to EU energy policy documents over seven decades and using insights from her theories to analyze who benefits, how costs and benefits are measured and policy effectiveness of energy efficiency policy. Building upon these discursive insights, I explore how the concept of energy efficiency can be broadened to take into consideration sociological and environmental issues.

## Energy efficiency policy research

Energy efficiency plays a major role in energy and environmental policy at all levels of government. It is a concept that straddles the science-policy interface, having both a strong scientific basis and also cachet as an effective policy tool. It is particularly popular as a non-controversial and low-cost strategy to reduce greenhouse gas emissions, especially in cases where the economy is not covered by binding carbon reduction targets or a price on carbon (Rosenow et al., 2018; Sorrell et al., 2011). Energy efficiency is an important part of European Union (EU) policy, with the European Commission having adopted the Energy Efficiency Directive (EED) 2012/27/EU. In December 2019, the European Commission reiterated its commitment to prioritize energy efficiency as part of its European Green Deal strategy to achieve carbon neutrality by 2050 (European Commission, 2019).

Energy efficiency research has been traditionally dominated by the ‘hard’ Science, Technology, Engineering, and Mathematics (STEM) disciplines (Dunlop, 2019). However, there are notable social sciences contributions. Some of the recent literature has focused on energy efficiency governance from an organizational perspective (e.g. Gupta and Ivanova, 2009; Jollands and Ellis, 2009) and on psychology and economics studies focusing on individual behaviors regarding energy consumption (e.g. Andrews and Johnson, 2016; Lopes et al., 2012). Much of this strand of literature in the EU is focused on attempting to solve major energy efficiency policy challenges, including off-track targets, monitoring challenges and non-compliance of Member States (e.g. Bertoldi, 2013; Harmsen et al., 2014). More widely in the energy and economics fields, there have been concerns raised about the perceived effectiveness of energy efficiency strategies in terms of energy savings (Herring, 2006) and reduction in energy demand (Sorrell, 2015). This is because it can be difficult to monitor and attribute energy savings to energy efficiency actions on the ground (Inhaber, 1997; Lopes et al., 2012).The issue is more complex given the conflicting evidence of the nature and extent of the rebound effect, whereby expected energy savings from energy efficiency measures may be spent elsewhere. For example, while energy savings may be made in the home with more energy efficient fluorescent lightbulbs, these gains can be offset if the consumer then decides to buy more lightbulbs than originally intended because they use less energy per unit. There are differing perspectives on the nature and extent of the rebound effect, such as what systemic changes occur and the amount of reduced energy savings (Ruzzenenti et al., 2019; Turner, 2013; van Den Bergh, 2011).

A number of social sciences and humanities scholars have sought to deconstruct the concept of energy efficiency as it relates to policy. A common critique is that energy efficiency has a narrow techno-economic focus that leaves policy ineffective and unbalanced in favor of economic interests; this is because it tends to separate humans from the natural world and stalls environmental action (Lutzenhiser, 2014; Shove, 2018). Similarly, asymmetrical power distribution in energy efficiency industry infrastructures, networks and regimes need more analysis to understand perverse effects and inequalities in society (Lutzenhiser, 2014; Winner, 1982). A related problem is the reductionist frame of individual behavior that tends to overlook qualitative solutions in favor of simplistic cause-effect relationships between exogenous factors (e.g. provision of information, price signals) and the actions of individual consumers (Labanca and Bertoldi, 2018). The effect of value judgments has been observed in a limited number of case studies where the choice of what to measure can focus on certain aspects and render others invisible. These studies have highlighted societal and environmental tradeoffs in the way that energy efficiency policies have been implemented, including an unfair economic cost burden on consumers (Owen, 1997), distraction away from alternative decarbonization measures such as renewables (Ruzzenenti and Wagner, 2018) and paradoxically increased energy use (Herring, 2000). Despite the substantial research into sociological aspects of energy efficiency (e.g. Herring, 1999; Lutzenhiser, 2014; Moezzi, 2000; Shove, 2018), there is a comparatively thin body of literature that observes EU energy efficiency policy through a historic lens. Furthermore, there is a gap in the literature that looks at how value judgments of the energy efficiency concept are manifest in historical policy in the EU and what effects these have sociologically. This article aims to fill these gaps by observing the dynamic evolution of the energy efficiency concept over time to understand underlying value judgments and tradeoffs in the way that it is conceptualized, defined and measured.

## Methods

The study looked at the EU because energy efficiency has been a significant focus for the European bloc over time and the EU encapsulates perspectives from various countries. Twenty policy documents were selected based on their relevance to energy efficiency policy, chosen to provide a comprehensive overview of policy between the 1950s and 2018. Although these documents were representative of the main themes that emerged in each decade, other documents (gray literature and scientific articles) were also assessed. The 1950s were selected as a start date to observe the characterization of energy efficiency in the post-war years before it took on a narrower meaning, and to see when, how and why energy efficiency became prominent in policy. A minimum of two documents were chosen from each decade, with criteria used to determine their relevance to policy based on the breadth and depth of their treatment of energy efficiency. This included the ways in which documents referred to energy efficiency, such as providing context, detail for planned actions and justification for actions taken. To ensure comparability, balance, and comprehensiveness of information, the documents analyzed included mainly EU directives provided by the Council of the European Union and ‘Communications’ written from the European Commission to the Council.^1^ Given that the EU has changed over time as an institution, documents were selected from earlier manifestations that included the Commission of the European Communities, European Coal and Steel Community, and European Atomic Energy Community.

An analytical framework of four code elements based on Stone’s (2012) discursive methodologies was developed: (1) symbols, or verbal languages, (2) numbers, numerical languages, (3) interests, and which actors are involved on different sides of a debate, and (4) facts, or information used to persuade. These codes were applied to the documents in three iterations of increasingly fine-grained analysis. The codes were analyzed, taking into consideration theoretical questions concerning efficiency as a motherhood issue: Who gets the benefits and bears the burdens of a policy? How should we measure the benefits and costs of a policy? What mode of organizing human activity is likely to yield the most effective results? The codes were then used to synthesize the discourse analysis below. It should be noted that the analysis is not intended as a comprehensive study of energy efficiency indicators and measures, but rather as a broad synthesis of conceptualizations of energy efficiency over time.

## The discourse of energy efficiency in EU politics

Discourse analysis across seven decades of energy efficiency policy documents in the EU illustrates significant variations in the concept of energy efficiency over time, including how it is defined and measured. The meaning of energy efficiency gradually becomes broader, more detailed and complex, as new concepts are attached to the term. While the quantitative characterization of energy efficiency as a ratio remains constant, the qualities and characteristics of the outputs in the equations change (such as, e.g. the substitute of energy output for gross domestic product or energy services). Another constant across the decades is that energy efficiency policy generally favors economic interests over social or environmental ones. This is revealed through language and in concrete policy actions. Below is a breakdown of the concept as it is defined and characterized in different eras.

### The nuclear era

Beginning in the 1950s, the documents describe concerns about limited natural resources, especially oil imports to Europe, following the 1956 Suez crisis, which disrupted the major shipping route through the Suez Canal, severely disrupting oil exports to Europe. In the 1960s, the threat is characterized as a coal industry in decline and over-dependence on energy imports. During these decades, nuclear energy is presented largely as the solution to energy supply problems. Efficiency in relation to energy is mentioned very little in the documents of the 1950s and 1960s. When efficiency is mentioned, it describes energy conversion processes^2^ in fairly simple terms that involve only energy inputs and outputs. In 1958, for example, ‘efficiency’ is described as ‘conversion efficiency’ (the ratio of the secondary energy obtained to the primary energy used to obtain it’) or ‘utilization efficiency’ (‘the ratio of effective energy obtained to total energy consumed’) (European Coal and Steel Community, 1958: 9). A 1950s document states the importance of being mindful about the use of limited natural resources, such as coal, oil, gas and peat. However, price concerns are seen to have a greater importance: ‘It is uncertain how low this “natural capital” will last. But still a more important point is how long it will be possible to go on securing energy supplies at present costs’ (European Coal and Steel Community, 1958: 6). Paradoxically, however, the same text does not state that atomic energy has any supply limits: ‘A growing population can only be assured of a high and rising standard of living if the quantities of energy available go on increasing’ (p. 5). Looking at interests, the main actors referred to include industry: coal plants, thermal power stations and transport sector. The texts of the 1950s refer to the human population as an aggregate entity, that is, ‘the population’ or ‘end-consumers’. There is no mention of experts or scientists, although predictions are made about future energy demand without stipulating how the figures were calculated and who calculated them.

### Oil crises

The 1970s are characterized by concerns about energy supplies in the wake of oil crises and high energy prices are said to threaten the standard of living and cohesion of the [EU] Community. Energy efficiency and nuclear energy are mentioned rarely. Instead, the main focus is on the ‘rational’ use of energy to avoid energy wastage: ‘The Commission has been examining the question of using energy in a more rational manner since the end of 1971, by which time it had become apparent that raw materials (including certain energy resources) were in short supply and often used wastefully’ (Council of the European Union, 1974: 4). Here, efficiency is characterized as a tactic to achieve a ‘rational use of energy’, which falls under the umbrella of ‘energy conservation’ more broadly. The aim of energy conservation in this context is to reduce energy use and achieve energy savings. Energy savings are measured according to an indicator of energy consumption. Efficiency is generally mentioned together with technical conversion processes, for example, efficiency of combustion, heat, lighting and power. In other cases, it is referred to vaguely, as an adjective (‘efficiently’) rather than as a noun (‘energy efficiency’) to describe less wasteful energy processes: ‘There are many techniques which could be employed in order to use energy more efficiently; the constraining factor is their economic viability’ (Council of the European Union, 1974: 6). Definitions at this time make clear that energy efficiency actions do not involve a reduction in energy output or comfort. A 1974 text explains that both energy efficiency and the reduction of consumption of non-useful energy aim ‘solely to reduce energy input while providing the consumer with the same level of output’. (Commission of the European Communities, 1974: 5). This is in contrast to a ‘demand restraint of useful energy’ which involves a ‘sacrifice on [the] consumer’s part’ (p.5). This sentiment is repeated in 2003: ‘Without reducing comfort or standards of living, it is therefore possible to reduce energy consumption by at least one-fifth at no extra net cost – and in many cases negative costs’ (Commission of the European Communities, 2003).

The actors mentioned during the 1970s are limited generally to the Administrative Authority (Commission of European Communities and Council) and experts and specialists who are mentioned as authority figures tasked with conducting assessments and solving problems. Experts and managers take prominence in the text as problem solvers. For example, national experts from the Energy Committee’s Working Party on the rational use of energy helped to formulate the action program. These specialists are also to be called upon to ‘organize an information drive for the general public’ (Commission of the European Communities, 1974: 10).

### Market rationalization

Following a global economic recession in the early 1980s, the problems in this decade are seen to be energy price increases, and the dependence of European countries on oil. Correspondingly, the documents show an increase in the use of economic and financial terms and concepts, linking them to energy efficiency. For example, the terms ‘competition’, ‘prices’ and ‘costs’ are used frequently, and ‘the market’ emerges in relation to energy efficiency and the rational use of energy. For example: ‘It is particularly important from the point of view of the rational use of energy that energy be priced with due regard for the market and costs’ (Council of the European Communities, 1985: 2). Energy efficiency is still seen as a tactic to achieve energy savings and a rational use of energy. However, mentions of the ‘rational’ use of energy and energy conservation decrease markedly, while mentions of energy efficiency increase. For example: ‘Efficient use of energy: This will be by far the most important factor influencing future energy consumption’ (Commission of the European Communities, 1985: 17). As it appears to decline in importance, the rational use of energy is referred to in a less defined and vague way, aligned with the virtue of prudence: ‘This Directive aims to preserve the quality of the environment and to ensure a prudent and rational utilization of natural resources’ (Council of the European Communities, 1993: 1). At times, the rational use of energy and energy efficiency are described together as if they were the same: ‘The increase in energy intensity during recent years and the reduced support for rational use of energy are symptomatic of the danger of complacency in energy efficiency during a period of stagnating or falling fossil fuel prices’ (Commission of the European Communities, 1987: 4).

The quantitative representation of energy efficiency in the 1980s adopts a new economic dimension with the energy intensity indicator, that is, units of energy per gross domestic product (GDP). In 1985, the document discusses the challenges of using various energy intensity indicators, which include the ratio between final energy demand and GDP as well as primary energy and GDP: ‘Neither of these measures provides a perfect basis for analysis of increased efficiency, since both can be influenced by changes in the structure of GDP’ (Commission of the European Communities, 1985: 25). The text explains that the indicator of ratio of final energy demand to GDP is chosen in order to keep measures simple, and because ‘the relationship between GDP and primary energy demand can be distorted by the rate of electricity penetration and the structure of electricity supply’ (Commission of the European Communities, 1985: 25). Correspondingly, in 1986, the Council adopts a resolution for a 20% improvement in energy intensity of final demand by 1995. Here, ‘benefits’ in relation to energy efficiency are mentioned for the first time: ‘Energy efficiency measures will also create new jobs and industrial activity and support environmental aims’ (Commission of the European Communities, 1985: 17).

The variety of different actors mentioned in the documents increases in this decade. There is less mention of undefined experts, and more of concrete industry figures, such as energy managers, savings advisors and consultants, as well as public institutions such as schools and universities. The ‘public’ is still referred to as an aggregate entity to be acted upon. For example, under the heading ‘Measures to encourage the rational use of energy’, the text states: ‘Information programs with a view to stimulating further public awareness on the efficient use of energy by advertising campaigns’ (Council of the European Communities, 1985: 2).

### Environmental awareness

The environment becomes a concern in the mid-1980s, when texts mention problems with pollution, such as from car exhaust fumes. Global warming is stated as a major problem in the 1990s and energy efficiency is linked to the reduction of carbon emissions. Energy efficiency becomes the main subject of legislative proposals for the first time in 1991, when the Council announces the ‘SAVE’ (Specific Actions for Vigorous Energy Efficiency) 5-year program. Thus, at this time, energy efficiency has gained more prominence with energy conservation and the rational use of energy only mentioned in reference to past policies. In 1993, energy efficiency is characterized as a means to achieve emissions reduction in the directive to ‘limit carbon dioxide emissions by improving energy efficiency’ (Council of the European Communities, 1993). The problem of global warming evolves into ‘climate change’ in the 2000s and 2010s. While these texts appear to focus on environmental concerns, evidence suggests that economic aspects are the main priority. In 1998, for example, the environment is mentioned as the most important issue, though very little detail regarding environmental objectives and actions is listed. The priority is ‘to underline the economic potential for energy efficiency’ (Commission of the European Communities, 1998: 1). In 1995, a 12% improvement in energy intensity of final consumption was achieved, falling 8% short from the 20% target. In 1998, a specific target was set to improve energy intensity of final consumption by an additional one percentage point per annum up to the year 2010.

Energy efficiency and technology are described as a ‘balanced approach’ solution to diffuse and harmonize conflicts between the opposing interests of the energy industry (growth, industry) and environmental interests (reducing greenhouse gas emissions). Hence, one major objective is described as: ‘The balanced pursuit of both energy and environmental aims, particularly through the use of the best available and cost-effective control technologies and through improvements in energy efficiency’ (Commission of the European Communities, 1985: 21). In 1998, the text vaguely refers to benefits: ‘Improved energy efficiency will lead to a more sustainable energy policy and enhanced security of supply, as well as to many other benefits’. (Commission of the European Communities, 1998: 1). In 1993, a link is made between energy efficiency and societal outcomes: ‘Improving energy efficiency in all regions of the Community will strengthen economic and social cohesion in the Community’ (Council of the European Communities, 1993).

In the 1990s, there is a notable focus on energy ‘services’, ostensibly to meet consumers’ wants and needs: ‘One of the most difficult institutional barriers to overcome today is the continued practice of selling energy in the form of kWh instead of as energy services such as heating and cooling, lighting and power. Services are invariably what the energy consumer actually wants, not energy for its own sake’ (Commission of the European Communities, 1998: 4). There is more emphasis on end-use consumers than in the decades before, while justifications are made to direct energy efficiency investments into the demand rather than the supply side: ‘Least-cost analyses show that investments in demand-side energy efficiency are often more cost-effective than production-side investments’ (Commission of the European Communities, 1998: 4). The language concerning actors has changed in the 1990s, with less emphasis on ‘educating’ people and more on ‘enabling’ them: ‘It is desirable that occupants of … buildings should be enabled to regulate their own consumption of heat, cold and hot water’ (Council of the European Communities, 1993: 1). The range of actors is more diversified, with most coming from the energy industry. These include energy service companies/industry, such as manufacturers, distributors, installers, industry associations, branch organizations, utilities and consumer organizations. Other actors include consumers, especially ‘end-use’ consumers.

### Markets as the solution

There is a strong economic focus in the 2000s and 2010s. The framing of market ‘barriers’ and ‘failures’ is prominent in these decades to explain challenges to be overcome. For example: ‘There is a clear need to improve the functioning of the energy market by removing barriers in order to allow market forces to allocate economic and natural resources effectively’ (Commission of the European Communities, 2003: 2). Later in the 2000s, solutions tend to be framed as part of the ‘energy efficiency market’. Following developments of the 1990s, the main aim is described as developing a market for energy end-use services, such as thermal comfort, domestic hot water, refrigeration, lighting comfort and motive power, and for the delivery of energy efficiency programs and other energy efficiency measures to end users. At this time, the documents tell a story of growth, unlike other decades, claiming that there is immense untapped market potential to realize energy efficiency benefits and improvements that, if solved, could lead to strong economic growth. Proposed solutions are often economic and technical in nature. For example, in 2006, actions outlined in the energy efficiency directive are to increase third-party financing for energy efficiency investments in the public sector and mechanisms to help consumers reduce their energy bills, such as changing billing practices for building occupants.

In 2003, the definition of ‘energy service’ is closely linked to technology: ‘Energy service: The physical amenity for energy end users derived from a combination of energy and energy using technology and, in certain cases, the operations and maintenance necessary to deliver the service (examples are indoor thermal comfort, lighting comfort, domestic hot water, refrigeration, product manufacturing, etc.)’ (Commission of the European Communities, 2003: 24).

While the actors mentioned during the 2000s and 2010s are increasingly diverse, they come predominantly from the energy efficiency industry and market – including investors, energy supply companies, energy distributors and retail suppliers, energy service companies, equipment installers and consultants. Consumers are still characterized as people to be acted upon: ‘For the market for energy efficiency to be tapped … the first task at hand will be to inform and convince consumers of the benefits of energy efficiency and to enable them to use energy-efficient technologies and energy efficiency measures effectively’ (Commission of the European Communities, 2003: 13).

### Energy efficiency benefits

From 2006 onwards, energy efficiency enjoys a headline status in policy with a number of directives referring exclusively to it as an end-goal, most notably in the directives 2006/32/EC on energy end-use efficiency and energy services and 2012/27/EU on energy efficiency. In 2006, the formal definition of energy efficiency gives a broader meaning to the output: ‘A ratio between an output of performance, service, goods or energy, and an input of energy’ (European Parliament and Council of the European Union, 2006: 67). In 2006, there are major changes in how the energy efficiency targets are measured. The 2006 energy intensity target is amended to measure final inland energy consumption – an indicative energy savings target for Member States to achieve a 9% saving over nine years. The directive acknowledges that the measure of final inland consumption ‘does not provide exact measurements at a detailed level, nor does it show cause and effect relationships between measures and their resulting energy savings. However, it is usually simpler and less costly and is often referred to as “energy efficiency indicators” because it gives an indication of developments’ (European Parliament and Council of the European Union, 2006: 114).

In the decade following 2010, the documents emphasize the benefits to be derived from energy efficiency improvements. In 2016, the text states: ‘A level higher than 27% energy efficiency in 2030 would bring higher benefits with regard to jobs and economic growth, security of supply, greenhouse gas emission reductions, health and environment’ (European Commission, 2016). In 2018, the text states that energy efficiency improvements will lead to improvements in the environment, air quality, public health, reduce greenhouse gas emissions, improve energy security, cut energy costs for households and companies and help alleviate energy poverty. This will lead to increased competitiveness, more jobs and increased economic activity throughout the economy and improve citizens’ quality of life. A ‘headline energy efficiency target’ is implemented in 2012 to reduce both primary and final energy consumption by 2020, and in 2018 the target is extended to 2030. In 2018, the ‘energy efficiency first’ principle is applied to energy policy, essentially placing a greater focus on energy efficiency policy options.^3^ The document states that the 2012 energy efficiency directive is an element to progress toward the Energy Union^4^ under which energy efficiency is to be treated as an energy source in its own right.^5^

In this decade, the range of actors mentioned has again increased, though still with a focus on actors in industry and the provision of energy services: ‘Cooperation with the private sector is important to assess the conditions on which private investment for energy efficiency projects can be unlocked and to develop new revenue models for innovation in the field of energy efficiency’ (European Parliament and Council of the European Union, 2018: 212). Further, there is a focus on ensuring the rights of final customers to fair billing practices and transparent information. These final consumers are defined as ‘natural or legal persons purchasing energy based on a direct, individual contract with an energy supplier’ (p. 214). The 2018 text also puts a focus on consultation of the directive annex with ‘experts’: ‘To ensure equal participation in the preparation of delegated acts, the European Parliament and the Council receive all documents at the same time as Member States’ experts, and their experts systematically have access to meetings of Commission expert groups dealing with the preparation of delegated acts’ (p. 215).

## Discussion

Stone quips that measuring efficiency is like ‘trying to pull yourself out of quicksand without a rope’ (Stone, 2012: 66). When it comes to efficiency policy, there is no firm ground. Policy objectives and energy efficiency policy are in a constant state of flux, evident in definitions, associations and measurements in each succeeding decade. On the one hand, energy efficiency is a ‘motherhood issue’ because conserving finite resources is common sense. Achieving savings, too, is a motherhood issue. However, the devil is in the details and the concept must be unpacked to see the underlying values at play. Who benefits from policies, how benefits and costs are measured and whether policies are in the end effective are questions that, when pulled apart, reveal values, and tradeoffs.

### Changes over time

Observing energy efficiency in a historical context shows how policy priorities and historical events are reflected in how energy efficiency is defined and applied in each decade. Perhaps it is no surprise that energy efficiency didn’t feature prominently in the energy policy in the 1950s through 1970s, given the dominance of nuclear energy promises and development.^6^ Nuclear energy was billed as the fuel that never runs out – a perspective far removed from the ‘no waste’ approach of energy efficiency and conservation policies that became popular later on. Energy efficiency in those decades was aligned with straightforward energy conversion processes of traditional fossil fuels along the energy chain, and actors were mainly energy industry actors such as coal and thermal power plants. The contrast between this conceptualization and the later definitions was stark. In the 1980s, the quantitative definition took on a monetary dimension as energy intensity, putting economic growth in focus in its link with energy production. Energy efficiency was mentioned more in the 1980s, and other strategies to reduce energy consumption mentioned less. This could indicate that the deep global recession of the early 1980s, together with the Three Mile Island accident of 1979 and Chernobyl crisis of 1986, boosted the influence of energy efficiency in policy. According to energy efficiency scholars, the shift toward energy efficiency policy in the 1980s was likely tied to a political shift toward economic priorities of free markets and capitalist expansion (Herring, 2006; Moezzi, 1998; Winner, 1982). Policy actions and discourses across the decades show that priorities generally lie in low-cost solutions. There are examples in each decade showing how cost considerations are prioritized over environmental and social concerns, both in the language used and in the specific policy actions outlined. The economic focus becomes stronger in the 1980s, with ‘the market’ featuring prominently in EU energy efficiency policy in later decades. This gradually narrows into the ‘energy efficiency market’. A shift toward a ‘market transformation’ globally^7^ is strongly represented in the language relating to ‘market barriers’ that are claimed to impede the realization of the full potential of energy efficiency.

### Rhetorical strategies of energy efficiency policy

The definitions and conceptualizations of energy efficiency are generally characterized as being value-neutral, technical and scientific. However, sometimes seemingly uncontroversial definitions and claims made regarding energy efficiency contain evaluative judgments, which act as persuasive strategies. For example, in 2006, energy efficiency is measured as a ratio between energy input and output of ‘performance, service, goods or energy’. This characterization of output is subjective, because ‘performance, service, good or energy’ differs in meaning from one person to another. However, not only were the output definitions linked to subjective ‘goods’, but they also evolved to contain built-in evaluative judgments. One major theme of the rhetoric across the decades is the idea of a ‘free lunch’ – that with energy efficiency, we get more for less or, in other words, something for nothing. For example, the energy efficiency definition assumes that there is no loss of comfort or convenience on the consumer’s part. In 1974, energy efficiency provides the consumer with ‘the same level of output’ (Commission of the European Communities, 1974: 5) in contrast to the ‘demand restraint of useful energy’, which involves a ‘sacrifice on [the] consumer’s part’ (p. 54). This assumption of stable comfort levels is echoed in 2003. As a consequence, energy efficiency is depicted as a more appealing option than the strategies to reduce energy that involve a restraint on demand and sacrifice on the consumer’s part. It is no surprise that energy efficiency was eventually adopted as the preferred strategy for energy reduction in EU policy.

The metaphor that depicts energy efficiency as ‘an energy source in its own right’ is another example of a free lunch promise. Here, the metaphor links energy efficiency (i.e. energy not wasted) with a tangible fuel. The metaphor is making something that never actually existed – energy that hasn’t been used – as valuable as tangible oil, gas or solar energy. Treating these sources as one and the same is methodologically problematic and somewhat misleading – using any given energy source has relative advantages and disadvantages (e.g. cost, location, carbon content), thus, it may not make sense to aggregate them as such. The energy efficiency as fuel metaphor resembles the ‘fuel that never runs out’ nuclear metaphor. Both depictions have an ethereal tone, as if energy efficiency and nuclear are perpetual energy machines or fountains of youth. But not all that glitters is gold – nuclear energy and efficiency come with hidden costs. Nuclear energy entails hefty upfront and maintenance costs, together with a long pollution life cycle and a risk of serious disaster. Energy efficiency involves investment that is either monetary, time or labor, and runs the risk that energy savings are not as high as expected. In this way, then, these act as stealth metaphors, ones of perpetual energy and false promise.

As energy efficiency becomes more prominent in the policy documents around the 1970s and 1980s, the various strategies to reduce energy consumption are often used interchangeably, hinting that they are honorifics given the same meaning. The terms ‘rational use of energy’ and ‘energy efficiency’ are often used together to generally mean less energy wastage and associated with the closely linked virtues of ‘rationality’, ‘prudence,’ and ‘efficiency’: ‘Rationality’ is defined as reaching your goal in the best possible way and ‘prudence’ means skill and good judgment in the use of resources. While various strategies to reduce energy consumption are well defined and delineated in the literature,^8^ it is recognized that these terms are often used ambiguously as if they were the same thing in policy and research (Herring, 2006; Moezzi, 2000; Oikonomou et al., 2009; Owen, 2000).^9^ Efficiency scholars argue that confusion over related terms may hamper efforts to effectively reduce consumption, given a lack of specificity of policy actions and targets for absolute energy consumption limits (Franco and Jorizzo, 2019; Goh and Ang, 2020). Therefore, communicating concepts relating to energy efficiency is important for effective policy implementation. Efforts were made in 2006, 2012, and 2018 to define energy efficiency and related concepts more accurately than they had been in previous decades. However, these more precise definitions became more complex, linked to a wide range of outputs and benefits. In addition, as the energy efficiency output was attributed to a longer list of benefits, it became a more effective rhetorical device. In the 1980s, energy efficiency promises a free lunch of societal benefits; the text states that energy efficiency measures would create new jobs and industrial activity, and would support environmental aims. Then, in the 1990s, energy efficiency improvements would supposedly bring stronger economic and social cohesion in the European Community. In the 2000s, the promise is largely one of economic growth. These promises are dramatically increased by 2018, when it is claimed that energy efficiency improvements will lead to such benefits as improved air quality, reduced greenhouse gas emissions, improved energy security, cuts to energy costs for households and companies, alleviation of energy poverty, increased competitiveness and economic activity, and more jobs and improvements for citizens’ quality of life (European Parliament and Council of the European Union, 2018: 210). Energy efficiency is a panacea to many of the region’s problems.

### What is left out?

A consistent assumption across the decades is that energy efficiency leads to energy savings and, therefore, reduces energy consumption. This provides the basis for later claims that energy efficiency leads to a reduction in greenhouse gas emissions. Given quantitative uncertainties, though, it cannot be assumed that energy efficiency actions automatically lead to energy savings (Pérez-Lombard et al., 2013). At certain times, the texts mention the issue of uncertainty with relation to indicators and forecasts, stating that energy efficiency measures and projections are not always accurate and should be treated solely as indicators. In the 1980s, for example, a detailed explanation of the weaknesses of certain indicators is discussed. However, in other decades, there is no mention of uncertainties in measures and quantification. It is surprising that no reference at any time is made to the rebound effect,^10^ despite decades of scientific literature on the topic. To be sure, this literature is contradictory and, as a consequence, the extent and nature of the rebound effect is unknown. Yet it is important not to ignore these uncertainties given that, despite decades of energy efficiency policy actions in Europe, the levels of primary and final energy consumption are similar to those in the 1990s (Eurostat, 2021), after peaking in 2006 (Tsemekidi Tzeiranaki et al., 2018) and global energy-related CO2 emissions are again nearing the historic peak set in 2018/2019 of 32.5 gigatonnes (Gt) (IEA, 2021). This is a general trend seen across the globe in recent decades, where, despite more efficient energy production and use, there has not been an absolute nor per capita reduction in energy use (Bertoldi, 2020). The inability to lower energy consumption levels globally has been put down to economic expansion, more production of goods, and greater demand for energy services, thanks to rising living standards in both developed and developing countries (Bertoldi, 2020).

One of the major problems with the quantitative expression of energy efficiency as a ratio is that energy conversion processes measure the rate of energy efficiency of a given conversion rather than measuring consumption overall. In other words, while one machine or appliance can be more efficient than another, it may still consume more energy in total if it is bigger, thus consuming more total energy. This is why, for example, as we purchase more efficient cars, lights and houses, energy consumption does not fall – because we buy bigger houses with more lighting and driving greater distances in more powerful cars. The energy efficiency ratio does not imply a cap or ceiling on consumption levels by default.^11^ This tends to obfuscate rising levels of consumption and shut down discussions about caps and limits on production and consumption. Limits are seen as increasingly important as awareness grows about the risks of overshooting our planetary boundaries. This problematic quantitative expression of energy efficiency is coupled with a narrative in the EU policy documents that aligns secure and low-cost energy with ideals of ‘progress’ and economic and social development. Such deterministic scientific narratives can be dangerous with the implication that the chosen policy actions are the only way to guarantee these outcomes and that lower levels of energy supply will threaten them. Furthermore, these narratives assume that everybody’s views on what ‘progress’ looks like are the same. Consequentially, this leaves little room for alternative narratives that might imagine a world with lower energy consumption – a positive result of different consumption practices and energy sources (see D’Alisa et al., 2014).

The framing of the later documents implies that ambitions to reduce energy consumption will be met by achieving energy efficiency targets, a significant shift from the earlier documents. Over seven decades, energy efficiency evolved from being a set of processes to become a target. Although the effectiveness of energy efficiency targets in Europe have been much debated,^12^ this article focuses on the conceptualizations behind the targets. That is, while attention in the media and in policy is placed on meeting targets and how high targets should be, discussions about how the targets are being defined and measured are less visible – as is what they mean from sociological perspectives. Issues such as limits, accepted consumption levels and quantitative uncertainty are discussed behind closed doors, if discussed at all. The question here is whether putting a focus on targets rather than methods is an effective policy approach. As Strathern states: ‘When a measure becomes a target, it ceases to be a good measure’ (Strathern, 1996). This point is even more important given that targets most likely do not accurately reflect progress due to energy efficiency actions in the first place (Herring, 1999). More emphasis is needed on how efficiency gains are measured. Measures could be altered to include, for example, the life-cycle analysis of products, to take into consideration the energy and pollution footprint of products along their whole lifetimes, rather than solely measuring energy efficiency at the consumer end. Measures could also be altered to take overall consumption more into account rather than simply the comparative efficiency levels of energy conversion processes. There are various alternative approaches to quantification that address problematic issues, including the interrelation between facts and values, inclusiveness and societal tradeoffs (see, e.g. Kovacic, 2018).

### Who benefits?

It is difficult to ascertain precisely who benefits from energy efficiency policy, especially given the complex and uncertain nature of energy efficiency benefits. Some beneficiaries are fairly straightforward to identify from the documents, and are unsurprising. The groups most mentioned over the decades in energy efficiency policy are increasingly those working in the energy efficiency industry and market, including energy efficiency technicians and experts, companies and organizations that undertake and lobby for energy efficiency actions. These groups are the prime benefactors of energy efficiency policies, given that various pieces of legislation bring with them calls for increased investment into energy efficiency ‘market’ and its ‘services’.

In later documents, the focus is increasingly on the ‘final consumer’, defined as ‘natural or legal persons purchasing energy based on a direct, individual contract with an energy supplier’ (European Parliament and Council of the European Union, 2018: 214). A great deal of detail is dedicated to regulations that ensure more transparent billing practices and comprehensive energy consumption information for final consumers. However, the largest consumer of final energy is the industry sector,^13^ rather than household or services sectors. It is curious, therefore, that more focus is not placed on energy reductions made by industry. This points to a need to better distinguish between types of final ‘consumers’.

A related point is that there is an omission of the role that consumers may play as ‘prosumers’. That is, the more recent texts fail to take into consideration that with changes to the relationship between consumers and providers (e.g. with renewable energy technologies), there is a greater role for consumers to play in generating their own energy and managing the supply to the grid (see Chappells and Shove, 2000). With recent easier access to renewable energy technology, we see a push by policymakers to open up the concept of consumers as ‘prosumers’ (European Commission and GfK Belgium consortium, 2017; European Parliament, 2016). The term assumes that consumers not only consume, but are part of the production process – generating and even selling electricity back to the grid. New technology, governance frameworks and community energy initiatives are offering new opportunities for citizens to get actively involved in energy matters, albeit in low numbers (Caramizaru and Uihlein, 2020).

The focus on energy efficiency services is at times justified by the perceived needs and desires of consumers: ‘Consumers want energy services, not energy in and of itself’ (Commission of the European Communities, 1998: 4). The consumers are already spoken for – treated as an aggregate entity, as if their needs and desires are one and the same. This is a problem across the decades, with ‘people’ referred to simply as consumers. In the 1960s and 1970s, nameless experts are depicted as the people who are best placed to make decisions on energy efficiency policy. Later on, markets and technology take a central role, with social concerns relegated to the periphery. In general, people are mostly characterized as energy consumers whose only interest is having energy services with the lowest cost and effort. Based largely on assumptions, this narrow characterization of citizens in energy efficiency policy fails to properly assess citizens’ needs and expectations. Missing are energy governance structures and organizational formats that are participatory, inclusive and mindful of the lived experiences of local people (Lennon et al., 2019). These include, for example, energy communities that generate their own renewable energy, with the proceeds going to community interests (for specific examples, see Caramizaru and Uihlein, 2020). The recognition of social issues, such as energy poverty in 2018, is a move in the right direction.

Technology plays a major role in how energy efficiency is conceptualized across the decades. Earlier policy sought to mitigate the conflict between energy and environmental interests with energy efficiency and technology as the ‘win-win’ solutions to solve disputes. Technology also has played a prominent role in defining energy efficiency in later decades, for example, as ‘energy services’.^14^ It could be that the lack of focus on people in energy policy is partially due to a prominent focus on seemingly uncontroversial solutions such as technology and energy efficiency. References to ‘consumer behavior’ become more prominent in later decades, effectively placing more onus on individual energy consumers to reduce their own energy consumption. However, focusing on individual energy consumption in this way presents an overly simplistic conceptualization of how we use energy (Labanca and Bertoldi, 2018). Such narrow frames can draw attention away from aspects that, if changed, could have more overall impact on energy consumption reduction – for example, by addressing power inequalities in energy regimes (Lutzenhiser, 2014; Winner, 1982), exploring more collective and non-commercial solutions (Wierling et al., 2018) and by better understanding how we use energy and why including issues to do with cultural norms and expectations (Shove and Walker, 2014). This includes, for example, more regulation on immensely powerful energy supply companies and more representation and inclusion of citizens in the process of generating and supplying energy.

### What can be done?

Given the problems outlined with the ways in which energy efficiency is defined and measured, what should be done with the concept of energy efficiency? For over a decade, academics and environmentalists, and now an increasing number of policymakers and NGOs, are calling for more focus on the concept of sufficiency, which implies a reduction in output. The difference between the two concepts is that energy efficiency reduces energy input while keeping the utility/services from energy constant, on the other hand, with sufficiency, energy consumption is reduced while the utility/technical service changes in quantity or quality (Princen, 2005; Thomas et al., 2015). Sufficiency doesn’t necessarily look to technology to reduce energy consumption, perhaps looking to community solutions and changes in energy consumption practices that reduce energy consumption overall: for example, opting to invest more in public transport and carsharing rather than focusing on more efficient cars for each family. This perspective mirrors Stone’s point that community and collaborative approaches that reflect societal dynamics are needed for meaningful policy outcomes rather than a narrow market focus.

Energy efficiency and sufficiency are not necessarily incompatible. In fact, they can be complementary as policy tools. Areas where the two concepts overlap include energy taxation, progressive energy prices as well as standards based on absolute consumption. However, sufficiency takes a different approach in other areas. An important part of sufficiency is to properly understand and mitigate drivers of unsustainable energy consumption, including capitalist production logics such as rapid innovation cycles and short product lifetimes, cultural norms for energy consumption (e.g. lower air conditioning temperatures and increasing tv-screen sizes). It focuses on alternative energy consumption practices that may involve less ‘comfort’ or less time saving for the consumer – such as drying clothes in the sun outside or putting limits on average dwelling floor area per person. However, ideas of ‘comfort’ or what levels of energy consumption are ‘enough’ are subjective and depend on individual and group culture and preferences. This is a challenging part of implementing sufficiency principles as subjective norms come into play (Darby, 2007). For example, how many cars in one family is enough? None, one, two or more? Should the floor surface area of houses be restricted? Coming to agreement on such topics will be a challenge going forward, with the need for better understandings of how to engage citizens in the issue of sufficiency and solve the challenge of how to define and understand needs, desires, and norms of energy use (e.g. Vadovics and Živčič, 2019).

## Conclusion

The definition and conceptualization of energy efficiency, while appearing to become more complex over time, has, in fact, narrowed. The concept is a useful political tool and, as a motherhood concept, it is a positively ambiguous euphemism for ‘good’ and ‘virtuous’, and its seemingly uncontroversial nature makes it difficult to criticize. It is no surprise that European institutions have invested heavily in the concept both in monetary and in policy terms. An increasing list of benefits has been attributed to energy efficiency, including its ability to provide energy services for less energy input and less cost, with no reduction in comfort. However, the historic and current conceptualization of energy efficiency omits important points, limiting its ability to solve contemporary complex problems such as reducing energy consumption and greenhouse gas emissions. Studies of energy efficiency need to be more focused on how it is measured and what tradeoffs occur as a result. The reconceptualization of energy efficiency using sufficiency principles is indicative of realizing this understanding. Historical analyses show that policies that imply a limit on energy services and consumption can easily be sidelined in favor of so-called ‘win-win’ solutions, namely energy efficiency and technological innovation, which do not necessarily lower energy consumption.

## Notes

1. However, in the earlier decades (1950s and 1960s), neither the directives nor communications dedicated to energy policy mention energy efficiency or efficiency in any detail. Therefore, strategic energy planning documents from these decades were analyzed to gain insight into the treatment of energy efficiency topics in those decades.

2. Primary energy is the total energy consumed, that is, the energy that is extracted from the earth as either oil, sun, wind, gas or coal. Secondary energy is the energy consumed at the consumer end. In between these two stages, the energy is converted, which involves a loss of energy. This conversion is called ‘conversion processes’.

3. The 2018 amendments to the 2012 directive explain that the ‘energy efficiency first principle’ means that ‘the Commission should ensure that energy efficiency and demand-side response can compete on equal terms with generation capacity. Energy efficiency needs to be considered whenever decisions relating to planning the energy system or to financing are taken. Energy efficiency improvements need to be made whenever they are more cost-effective than equivalent supply-side solutions’.

4. The energy union strategy (COM/2015/080) was a priority of the European Commission (2019) aimed at building an energy union that gives EU consumers – households and businesses – secure, sustainable, competitive and affordable energy.

5. In light of the stronger targets set for energy efficiency for 2020 and 2030, the Commission states that energy efficiency should be treated as an energy source in its own right, representing the value of energy saved (European Commission, 2015). This appears to stem from Commission research showing that by 2030, more energy will be saved than the amount of energy consumed deriving from oil. Using this justification, the Commission states that energy savings can be considered as ‘an energy source in its own right’. This depiction may have been inspired by the prominent 2013 IEA report (Oecd/iea, 2013) that found the energy savings from efficiency measures taken over the longer term exceeded the output from any other single fuel source in 11 countries. Here, the IEA uses the data to justify not only that energy efficiency is an energy resource, but could be referred to as ‘the first fuel’ (OECD IEA, 2013: 3).

6. Although ‘energy efficiency’ was not specifically mentioned during these decades, efficiency as it relates to energy was mentioned. However, it was conceptualized in a different way and mentioned rarely.

7. The ‘market transformation’ refers to a policy objective to promote energy efficient technologies in the marketplace. The intervention aims to alter market behavior by removing barriers and capitalizing on opportunities to internalize cost-effective energy efficiency practices.

8. ‘Energy efficiency’ is thought of as reducing energy input whilst keeping energy services constant, while ‘energy conservation’ is often thought of as reducing energy input by reducing energy services (Labanca and Bertoldi, 2018). The rational use of energy is a broad set of actions to reduce energy consumption that include energy efficiency, energy conservation, energy waste reduction, behavior change and the use of renewable energy sources (Franco and Jorizzo, 2019).

9. In Europe, ‘energy conservation’ was the preferred term used in policy until ‘energy efficiency’ replaced it as the policy action of choice to reduce energy consumption (Owen, 2000). On the other hand, in the U.S., The term ‘energy conservation’ fell out of favor in the 1970s, when it was aligned with ‘pain’ and ‘sacrifice’ during the Carter era and oil crises (Moezzi, 2000). It is possible that the ‘rational’ use of energy was popular because of the influence of the French language in EU policy at the time – the ‘rational’ use of energy was a favored term in French that aligned with the idea of ‘energy efficiency’ (*utilisation rationnelle de* l’*énergie*).

10. In 2017, a question was made by parliamentarian Philippe Lamberts (Verts/ALE) about the rebound effect (https://www.europarl.europa.eu/doceo/document/P-8-2017-007339_EN.html), with a reply stating that the EED impact assessment takes into consideration the rebound effect, with a lower (21%) and a higher (43%) value. It is not clear, however, how the directive handles the uncertainty of the rebound effect, given these two possible scenarios (https://www.europarl.europa.eu/doceo/document/P-8-2017-007339-ASW_EN.html).

11. It should be noted that EU headline targets are now measured in energy consumption. In other words, the energy efficiency itself is not measuring energy efficiency per se, but the intended consequence of energy efficiency measures – energy savings. The point here is that it is difficult to attribute energy efficiency actions at the micro level with measured energy savings at an aggregated, macro level (Herring, 1999). Further, Member States can choose to measure their efficiency improvements using the energy intensity indicator. Authors have explained the problematic nature of the indictor, mainly pointing to the fact that it often cannot accurately measure underlying technical efficiency nor externalities such as the energy intensity of imported energy. There is thus a common trend in Europe whereby while energy intensity has been reduced, energy consumption has increased (Bertoldi, 2020). Yet, European countries can still choose which indicator they would like to use, including energy intensity, to demonstrate energy efficiency improvements.

12. According to Jollands (2012), for example, a concern is that setting targets can be an exercise in political publicity – particularly when the burden for achieving the target rests on future politicians. Further, he argues, putting effort into energy efficiency targets can distract policy makers from actually delivering on energy efficiency improvements. One significant problem in recent years has been that European Commission has not been able to resolve how to monitor progress against national energy efficiency targets after several years of trying, partly due to the fact that ‘saved energy’ is intangible and thus difficult to measure.

13. In 2019, the industry sector (32% of final energy consumption) consumes the most energy, followed by the transport sector (28 %), households (24%), services (12%) and agriculture and forestry (3%) (https://ec.europa.eu/eurostat/cache/infographs/energy/bloc-3a.html)

14. In 2003, energy service is defined as: ‘The physical amenity for energy end users derived from a combination of energy and energy-using technology and, in certain cases, the operations and maintenance necessary to deliver the service (examples are indoor thermal comfort, lighting comfort, domestic hot water, refrigeration, product manufacturing, etc.) (Commission of the European Communities, 2003: 24)
